# Fecal Microbiota Transplantation as a Rescue Therapy for Refractory *Clostridioides difficile* Infection in a Patient With Lupus

**DOI:** 10.1155/crdi/7694915

**Published:** 2026-09-29

**Authors:** Miguel Alejandro Pinzón, Juan Felipe Betancur, Andrea Paola Sierra Bolaño, Julio Andrés Boada-Garnica, Juan Felipe Llano, Beatriz Ramírez

**Affiliations:** ^1^ Department of Infectious Diseases, Clínica Medellín Grupo Quirónsalud, Medellín, Colombia; ^2^ Internal Medicine Research Unit, Centro de Investigaciones Clínicas SURA, Medellín, Colombia; ^3^ Clinical Research Department, Clínica Medellín Grupo Quirónsalud, Medellín, Colombia; ^4^ Inpatient Physician, Clínica Medellín Grupo Quirónsalud, Medellín, Colombia

**Keywords:** *Clostridioides difficile* infection, fecal microbiota transplantation, fulminant infection, refractory infection, rescue therapy, systemic lupus erythematosus

## Abstract

*Clostridioides difficile* infection (CDI) remains a major clinical challenge, particularly when it is refractory or recurrent. We describe a 68‐year‐old woman with systemic lupus erythematosus and other comorbidities who developed fulminant refractory CDI, characterized by ileus and persistent symptoms despite a course of oral vancomycin. She presented with abdominal pain, bilious vomiting, and high‐volume diarrhea. Stool testing detected *C. difficile* toxins A and B, and imaging showed bowel dilatation, mucosal thickening, and lymphadenopathy. Despite 18 days of oral vancomycin and intravenous metronidazole, she showed no clinical improvement. A single fecal microbiota transplantation (FMT) was therefore performed as rescue therapy. Diarrhea resolved completely within 3 days, with no CDI recurrence or FMT‐related adverse events during 12 months of follow‐up. This case illustrates the potential role of FMT as rescue therapy for fulminant refractory CDI in selected immunocompromised patients. Careful donor screening and close clinical monitoring remain essential, and further research is needed to define the long‐term safety of FMT in patients with autoimmune diseases.

## 1. Introduction

*Clostridioides difficile* (*C. difficile*) is an obligate anaerobic, gram‐positive, spore‐forming bacterium and an important cause of healthcare‐associated diarrhea. Its toxins A and B disrupt intestinal epithelial function and cause *C. difficile* infection (CDI). Clinical severity ranges from mild, self‐limited diarrhea to pseudomembranous colitis, sepsis, toxic megacolon, intestinal perforation, and death.

The incidence and severity of CDI have increased in recent decades, partly because of widespread antibiotic exposure. Although antimicrobial therapy remains the cornerstone of treatment, it also disrupts the normal intestinal microbiota and creates conditions that favor *C. difficile* proliferation and recurrence.

This report describes fulminant refractory CDI in a patient with systemic lupus erythematosus (SLE) who was successfully treated with fecal microbiota transplantation (FMT). It highlights the diagnostic and therapeutic challenges of CDI in an immunocompromised host and the potential role of FMT as rescue therapy.

## 2. Case Presentation

A 68‐year‐old woman with hypertension, obesity, deep vein thrombosis without antiphospholipid syndrome, immune thrombocytopenic purpura, and SLE was receiving prednisone 5 mg/day, hydroxychloroquine 400 mg/day, and rivaroxaban 10 mg once daily. She had previously been hospitalized for CDI and treated with a 14‐day course of oral vancomycin. Despite completing treatment, she returned with lower abdominal pain, copious bilious vomiting, and high‐volume diarrhea, with up to 20 bowel movements per day. Initial laboratory tests showed metabolic acidosis without hypoxemia (base excess, −4.5), blood urea nitrogen 23 mg/dL, creatinine 1.21 mg/dL, C‐reactive protein 0.3 mg/dL, hemoglobin 12.1 g/dL, hematocrit 33.1%, white blood cell count 14,020/μL, neutrophils 86.3%, lymphocytes 6.7%, monocytes 6.0%, and platelet count 319,000/μL, without electrolyte abnormalities. Stool examination showed soft stool, occult blood, and no mucus, leukocytes, or erythrocytes. The FilmArray gastrointestinal panel detected *C. difficile* toxins A and B. *Campylobacter*, *Plesiomonas shigelloides*, *Salmonella* spp., *Vibrio cholerae*, other *Vibrio* spp., *Yersinia enterocolitica*, enteroaggregative *Escherichia coli*, enteropathogenic *E. coli*, enteroinvasive *E. coli*/*Shigella*, and Shiga toxin‐producing *E. coli* were not detected. *Cyclospora cayetanensis*, *Cryptosporidium* spp., *Entamoeba histolytica*, and *Giardia lamblia* were also not detected.

The patient experienced a prolonged and complex clinical course characterized by marked abdominal distension and ileus. Because ileus is a defining feature of fulminant CDI, the episode was classified as fulminant refractory CDI despite the absence of hypotension, shock, or toxic megacolon. Treatment consisted of oral vancomycin 250 mg every 6 h and intravenous metronidazole 500 mg every 8 h. Endoscopic tube placement was also required. Contrast‐enhanced abdominal computed tomography (CT) showed dilatation of both small‐ and large‐bowel loops, with air‐fluid levels and liquid stool. There was no intestinal pneumatosis or free intraperitoneal air. Fluid was present in the paracolic gutters and perihepatic and perisplenic spaces, with edema of the periabdominal soft tissues. A small hiatal hernia and gallstones were also identified (Figure 1). Contrast‐enhanced abdominal magnetic resonance imaging showed moderate circumferential mucosal thickening at the rectosigmoid junction and upper rectum, with restricted diffusion and proximal dilatation of the intestinal loops. Perisigmoid and right common iliac chain lymphadenopathy was also present.

**FIGURE 1:**
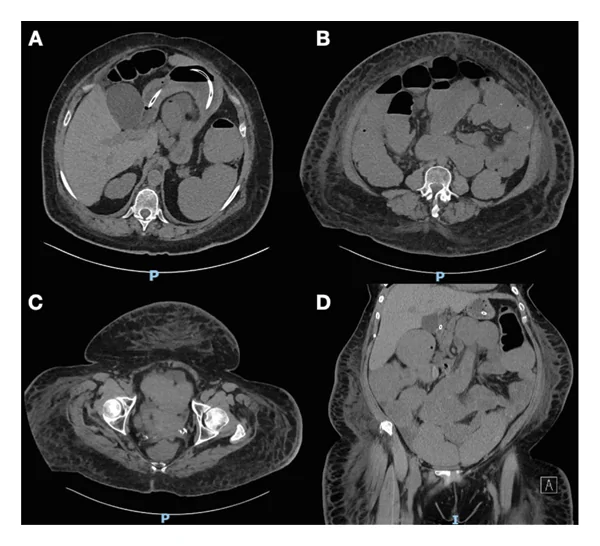
Contrast‐enhanced abdominal computed tomography showing dilatation of small‐ and large‐bowel loops, with air‐fluid levels and intraluminal liquid content.

After 18 days of antimicrobial therapy without clinical improvement, the patient underwent a single FMT session as rescue therapy. The procedure followed internationally accepted recommendations for donor screening, stool preparation, and FMT administration. The donor was a healthy adult first‐degree relative with no recent antibiotic exposure or history of gastrointestinal, autoimmune, or infectious disease. Clinical and laboratory screening was negative for human immunodeficiency virus, hepatitis B virus, hepatitis C virus, syphilis, *C. difficile* toxins A and B, enteric parasites, and multidrug‐resistant organisms.

Approximately 50 g of fresh stool was collected on the day of the procedure, diluted in 200 mL of sterile normal saline, homogenized, and filtered through sterile gauze to produce a uniform fecal suspension (Figure 2). The recipient underwent standard bowel preparation, and antimicrobial therapy was discontinued 48 h before FMT. The suspension was administered slowly as a rectal retention enema and retained for approximately 3 h.

**FIGURE 2:**
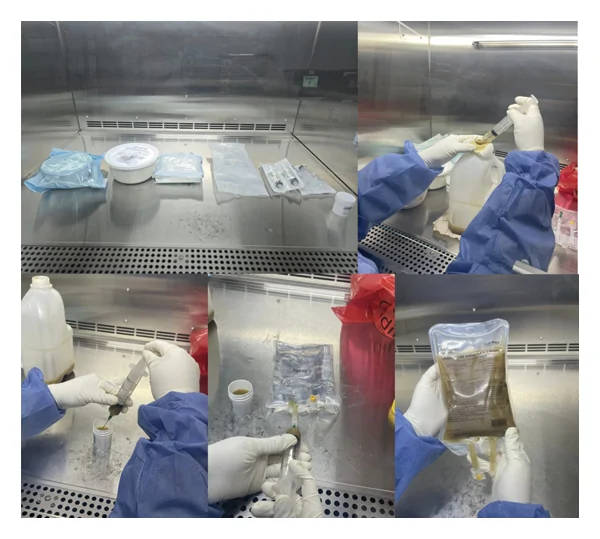
Preparation of the fecal suspension for microbiota transplantation.

The procedure was well tolerated, with no immediate or early procedure‐related complications. Diarrhea resolved completely within 3 days after FMT. During 12 months of clinical follow‐up, no CDI recurrence or procedure‐related adverse events were documented.

## 3. Discussion

*C. difficile* is a strictly anaerobic, gram‐positive, spore‐forming bacterium. Its toxins A and B impair intestinal epithelial function and lead to CDI [1]. The widespread use of antibiotics has contributed to the growing burden of CDI, making it a major public health concern [1].

Clinical manifestations of CDI range from mild, self‐limited diarrhea to pseudomembranous colitis, sepsis, toxic megacolon, intestinal perforation, and death. Antimicrobial therapy is the mainstay of treatment [1]. Current international guidelines recommend fidaxomicin as the preferred therapy for initial and recurrent CDI because it is associated with lower recurrence rates than vancomycin. Oral vancomycin remains an acceptable alternative when fidaxomicin is unavailable. In this case, fidaxomicin was unavailable in Colombia at the time of treatment; therefore, oral vancomycin and intravenous metronidazole were used according to local availability and institutional practice [1].

In this case, clinically and radiologically documented ileus supported classification as fulminant CDI, despite the absence of hypotension, shock, or toxic megacolon. Moreover, because the patient did not achieve complete clinical resolution or experience a symptom‐free interval after the initial course of oral vancomycin, the infection was classified as refractory rather than recurrent.

Recurrent CDI is a considerable clinical challenge and occurs in approximately 20% of patients [1]. The risk of a subsequent recurrence may reach approximately 40%–45% [1]. International guidelines favor fidaxomicin over a standard course of vancomycin for recurrent CDI. FMT is an established microbiota‐restoration strategy for recurrent CDI and may also be considered as rescue therapy in selected patients with severe, fulminant, or refractory disease.

In patients with CDI complicated by colonic perforation or toxic megacolon, surgical intervention, including colectomy, may be required [1].

*C. difficile* infection is clinically important in patients with SLE. Infections, including CDI, may trigger SLE exacerbations, cause severe complications, or mimic gastrointestinal vasculitis [2, 3]. Severe CDI has been associated with poor outcomes in individual patients with SLE, including those receiving corticosteroids or immunosuppressive agents such as rituximab and cyclophosphamide [2].

In an analysis of 29,549 patients hospitalized with CDI in the National Inpatient Sample, 332 had SLE. Patients with SLE were younger (mean age, 53.8 vs. 66.4 years), were predominantly female (91.0% vs. 63.9%), and were more frequently obese (14.6% vs. 9.7%) than those without SLE. They were less likely to be White (55.7% vs. 78.4%), had a lower Charlson Comorbidity Index (3.8 vs. 4.2), and had lower unadjusted mortality (0.3% vs. 1.8%). Length of stay and hospital charges did not differ significantly. After adjustment, SLE was associated with lower odds of hypotension or shock (adjusted odds ratio, 0.45; 95% confidence interval, 0.23–0.88). The adjusted odds of renal failure, ileus, colectomy, and mortality did not differ significantly; megacolon was not analyzed because of its low prevalence [4].

FMT restores intestinal microbial diversity and colonization resistance and is highly effective for recurrent CDI, with reported cure rates approaching or exceeding 90% in selected series [5–8]. Evidence also supports its potential use as rescue therapy for severe, fulminant, or refractory CDI [5, 6]. In a prospective Japanese case series of patients with refractory CDI, all participants achieved clinical resolution after a single FMT, with no adverse events during 3 months of follow‐up [9]. FMT may therefore offer a nonsurgical option for selected patients who do not respond to antimicrobial therapy or are poor surgical candidates [5].

The safety of FMT in immunocompromised patients remains an important concern. Although FMT is generally considered safe, evidence in severely immunocompromised populations is limited [8]. A published case demonstrated successful FMT for refractory CDI in a severely immunocompromised patient, suggesting that this approach may be feasible in carefully selected cases [10]. Rigorous donor selection, standardized preparation, and close postprocedure monitoring are essential to minimize infectious and other risks [7, 8, 11].

Although FMT is used primarily for CDI, it has also been investigated in conditions such as inflammatory bowel disease [11]. Its potential to modulate host immunity and restore intestinal microbial balance has prompted interest in broader applications; however, further research is required to establish its safety and efficacy in autoimmune diseases [11].

FMT is not currently a first‐line treatment for CDI [12]. Further research is needed to optimize its preparation and administration, standardize clinical protocols, and establish long‐term safety, particularly in immunocompromised patients and those with autoimmune diseases [11, 12]. The present case supports the potential role of FMT as carefully selected rescue therapy for fulminant refractory CDI when conventional antimicrobial treatment fails.

## 4. Conclusion

This case demonstrates the successful use of FMT as rescue therapy for fulminant refractory CDI in a patient with SLE receiving immunomodulatory therapy. Ileus supported classification as fulminant CDI, whereas persistent symptoms without a symptom‐free interval after antimicrobial treatment supported classification as refractory rather than recurrent infection. Diarrhea resolved completely within 3 days after a single FMT session, with no CDI recurrence or procedure‐related adverse events during 12 months of follow‐up. Careful donor screening and close clinical monitoring are essential when FMT is considered in immunocompromised patients.

## Author Contributions

All authors contributed to the conception, design, data collection, and interpretation of this case. Juan Felipe Betancur, Miguel Alejandro Pinzón, Andrea Paola Sierra Bolaño, Julio Andrés Boada‐Garnica, Juan Felipe Llano, and Beatriz Ramírez drafted the manuscript and critically revised it for important intellectual content.

## Funding

The authors received no financial support for the research, authorship, or publication of this article.

## Disclosure

All authors approved the final version and agreed to be accountable for all aspects of the work.

## Ethics Statement

All procedures were conducted in accordance with the ethical standards of the responsible institutional and national committees on human experimentation and with the Declaration of Helsinki, as revised in 2008.

## Consent

Written informed consent was obtained from the patient for publication of this case report and the accompanying images. All identifying information was anonymized to protect patient confidentiality.

## Conflicts of Interest

The authors declare no conflicts of interest.

## Supporting Information

Additional supporting information can be found online in the Supporting Information section.

## Supporting information


**Supporting Information** CARE Checklist.

## Data Availability

The data that support the findings of this study are available from the corresponding author upon reasonable request.
