# Supplementary material for: Fecal Microbiota Transplantation as a Rescue Therapy for Refractory Clostridioides difficile Infection in a Patient With Lupus
Source: Case Rep Infect Dis. 2026 Sep 29;2026:7694915. doi: 10.1155/crdi/7694915 (PMC13621822; doi:10.1155/crdi/7694915)
Supplement: Supplementary file 1 [file CRDI-2026-7694915-s001.docx]

**CARE Checklist**

**Title of Manuscript:** *Fecal Microbiota Transplantation as a Rescue Therapy for Refractory Clostridioides difficile Infection in a Patient with Lupus*

| **CARE Item** | **Description** | **Reported on page(s)** |
| --- | --- | --- |
| **1. Title** | The words "case report" together with the phenomenon of greatest interest | Page 1 |
| **2. Key words** | 2–5 key words identifying the diagnosis or intervention | Page 3 |
| **3a. Abstract – Introduction** | What is unique about this case and why is it important? | Page 2 |
| **3b. Abstract – Clinical findings** | Main symptoms and clinical findings | Page 2 |
| **3c. Abstract – Diagnosis, intervention and outcomes** | Diagnosis, intervention, and outcomes | Pages 2–3 |
| **3d. Abstract – Conclusion** | Main lessons learned | Page 3 |
| **4. Introduction** | Background and relevant literature | Pages 3–4 |
| **5a. Patient information** | Demographic characteristics | Page 4 |
| **5b. Main symptoms** | Chief complaints | Page 4 |
| **5c. Medical, family and psychosocial history** | Relevant medical history, medications and comorbidities | Page 4 |
| **6. Clinical findings** | Relevant physical examination and clinical findings | Pages 4–5 |
| **7. Timeline** | Chronological timeline of the case | Pages 4–5 |
| **8a. Diagnostic assessment** | Diagnostic methods | Pages 4–6 |
| **8b. Diagnostic challenges** | Financial, cultural or other barriers | Not applicable |
| **8c. Diagnostic reasoning** | Differential diagnosis and reasoning | Pages 5–6 |
| **8d. Prognostic characteristics** | Staging or severity classification when applicable | Pages 5–6 *(Fulminant CDI clarified in revised manuscript)* |
| **9a. Therapeutic intervention** | Type of intervention | Pages 5–6 |
| **9b. Administration** | Dose, duration, route | Pages 5–6 |
| **9c. Changes in intervention** | Modifications with rationale | Pages 5–6 |
| **10a. Follow-up and outcomes** | Clinical course and follow-up | Page 6 |
| **10b. Follow-up test results** | Outcomes and clinical evolution | Page 6 |
| **10c. Adherence and tolerability** | Intervention adherence and adverse events | Page 6 |
| **10d. Adverse events** | Unexpected events | Page 6 (none observed) |
| **11a. Discussion** | Strengths and limitations | Pages 6–10 |
| **11b. Literature review** | Relevant literature | Pages 6–10 |
| **11c. Scientific rationale** | Interpretation of findings | Pages 6–10 |
| **11d. Take-away lessons** | Main lessons from the case | Page 10 |
| **12. Patient perspective** | Patient's own perspective | Not available |
| **13. Informed consent** | Written informed consent obtained | Page 10 |
